# Effect of pneumovesicoscopic cohen surgery with adjustable suspension technique through the urethra on the treatment of primary vesicoureteral reflux disease in infants

**DOI:** 10.1186/s12893-023-01996-7

**Published:** 2023-04-28

**Authors:** Xu Cui, Wen-Hua Huang, Yun-Jin Wang, Liu Chen, Jian-Qin Zhang, Chao-Ming Zhou

**Affiliations:** 1 Department of Pediatric Surgery, Fujian Maternity and Child Health Hospital College of Clinical Medicine for Obstetrics & Gynecology and Pediatrics, Fujian Medical University, Fuzhou, 350001 P. R. China; 2 Fujian Children’s Hospital (Fujian Branch of Shanghai Children’s Medical Center), College of Clinical Medicine for Obstetrics & Gynecology and Pediatrics, Fujian Medical University, Fuzhou, China; 3 College of Clinical Medicine for Obstetrics & Gynecology and Pediatrics, Fujian Medical University, Fuzhou, 350001 P. R. China

**Keywords:** Pneumovesicoscopic Cohen surgery, Adjustable suspension technique, Natural channel, Primary vesicoureteral reflux disease, Infants

## Abstract

**Objective:**

The objective of this study was to evaluate the safety and efficacy of pneumovesicoscopic Cohen surgery with an adjustable suspension technique through the urethra for the treatment of primary vesicoureteral reflux disease in infants.

**Methods:**

This study retrospectively analysed the clinical data of 31 infants who underwent pneumovesicoscopic Cohen surgery with an adjustable suspension technique through the urethra in our hospital from January 2019 to December 2020. We also collected the clinical data of 29 infants who underwent open Cohen surgery in our hospital from January 2015 to December 2018 as a control variable. The clinical efficacy of the two groups was compared.

**Result:**

All pneumovesicoscopic Cohen surgeries were successfully completed and no patients were converted to open surgery. The amount of bleeding, duration of postoperative analgesia, duration of postoperative haematuria, incision size and length of hospital stay in the pneumovesicoscopic surgery group were significantly lower than those in the open surgery group (P < 0.05). The operation time of the pneumovesicoscopic surgery group was significantly longer than that of the open surgery group (P < 0.05). Both groups were followed for six months after surgery. At the 6-month follow-up time, there were no significant differences in the degree of hydronephrosis, renal scarring, renal atrophy, glomerular filtration rate, or KIM-1 and MCP-1 expression between the two groups (P > 0.05).

**Conclusion:**

Pneumovesicoscopic Cohen surgery with an adjustable suspension technique through the urethra for the treatment of primary vesicoureteral reflux disease in infants was safe and effective. This procedure had the advantages of less trauma, quick recovery and good cosmetic effects.

## Introduction

Primary vesicoureteral reflux (VUR) disease is the most common reason for recurrent febrile urinary tract infection, reflux nephropathy, renal scarring and end-stage renal disease in children [1–3]. The incidence of VUR in children is 0.4-1.8% and shows an increasing trend year by year [4, 5]. Approximately 30% of VUR cases cause hypertension and chronic renal failure in children and it is the most common cause of childhood end-stage renal disease [6]. Therefore, early detection and resolution of reflux and renal scar formation are the most important principles of VUR [6, 7]. Anti-reflux surgery is the main treatment for VUR. Open ureterovesical reimplantation (Cohen surgery), with a high surgical success rate, is widely used and is the “gold standard” in anti-reflux surgery [8, 9]. However, it has disadvantages such as a large incision, extensive trauma, and hypertrophic scarring.

With the rise of microinvasive aerocystoid techniques, pneumovesicoscopic reimplantation has also been gradually accepted by paediatric urologists and has achieved good therapeutic effects [10–13]. However, the operation is difficult, especially for infant patients. Because of the small space operation, the operation is more challenging, technically demanding, and the learning curve is longer. In addition, there are few clinical studies on vesicoureteral reimplantation in infants. In our centre, we modified the technique of pneumovesicoscopic Cohen surgery and performed pneumovesicoscopic Cohen surgery with an adjustable suspension technique through the urethra for the treatment of primary vesicoureteral reflux disease in infants. This study summarized our surgical experience and evaluated the safety and efficacy of the operation.

## Methods

### Patients

This study retrospectively analysed the clinical data of 31 infants who underwent pneumovesicoscopic Cohen surgery with an adjustable suspension technique through the urethra in our hospital from January 2019 to December 2020. We also collected the clinical data of 29 infants who underwent open Cohen surgery in our hospital from January 2015 to December 2018 as a control. The clinical efficacy of the two groups was compared.

Many studies have shown that KIM-1 and MCP-1 in urine can reflect the severity of kidney injury and fibrosis [14–16]. Before surgery and 6 months after surgery, urological ultrasound was routinely performed to investigate the anteroposterior diameter of hydronephrosis and distal ureter diameter. Additionally, a voiding cystourethrography was used to investigate the grade of reflux alongside dimercaptosuccinic acid to evaluate the formation and grade of renal scar, and renal function status. The levels of KIM-1 and MCP-1 in morning urine were detected to evaluate the progression of renal injury and fibrosis.

The inclusion criteria were infants with primary VUR undergoing Cohen surgery. The exclusion criteria were the following: (1) Complicated with other urinary tract malformations, including posterior urethral valve, urethral stricture, neurogenic bladder, ureteropelvic junction stenosis, etc. (2) Complicated with other serious diseases, such as congenital heart disease, severe liver and kidney dysfunction, etc. (3) The clinical data was incomplete. (4) Age less than 6 months or weight less than 6 kg. (5) Parents of patients refused to participate in the study.

### Surgical technique

#### Pneumovesicoscopic cohen surgery with an adjustable suspension technique through a natural channel

**Fig. 1:**
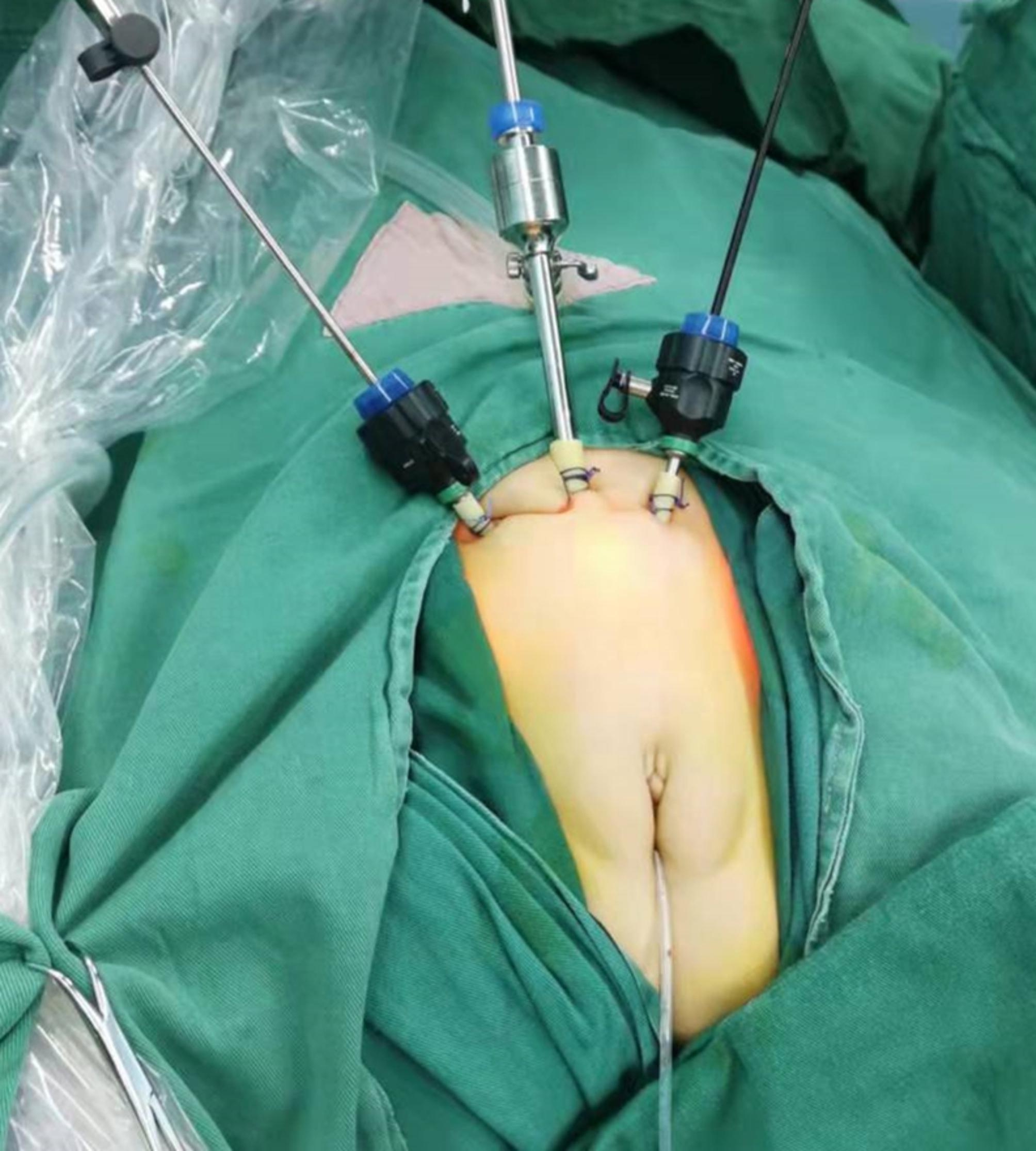
The position of Trocars and the fixation of Trocars

The patients were placed in the lithotomy position with two legs horizontally abducted. Routine disinfection towels were placed. The F8 Storz 30 cystoscope was inserted into the bladder and bladder pressure was set at 14 mmHg. We set two 2 − 0 absorbable stitches that penetrated into the antetheca of the bladder and out from the penetrated point at approximately 3 cm below the middle of the navel. The suture was left unknotted. The other 2 sutures were set on both sides approximately 2 cm away from the first puncture point at the same level. The indwelling stitches were lifted, and then a 5 mm trocar was placed into the bladder from the middle of the first two sutures. Then, the other two 3 mm trocars were set in the same way in the position of the indwelling suture. The suture was then tied down to fix the trocar to the skin (Fig. 1).

**Fig. 2:**
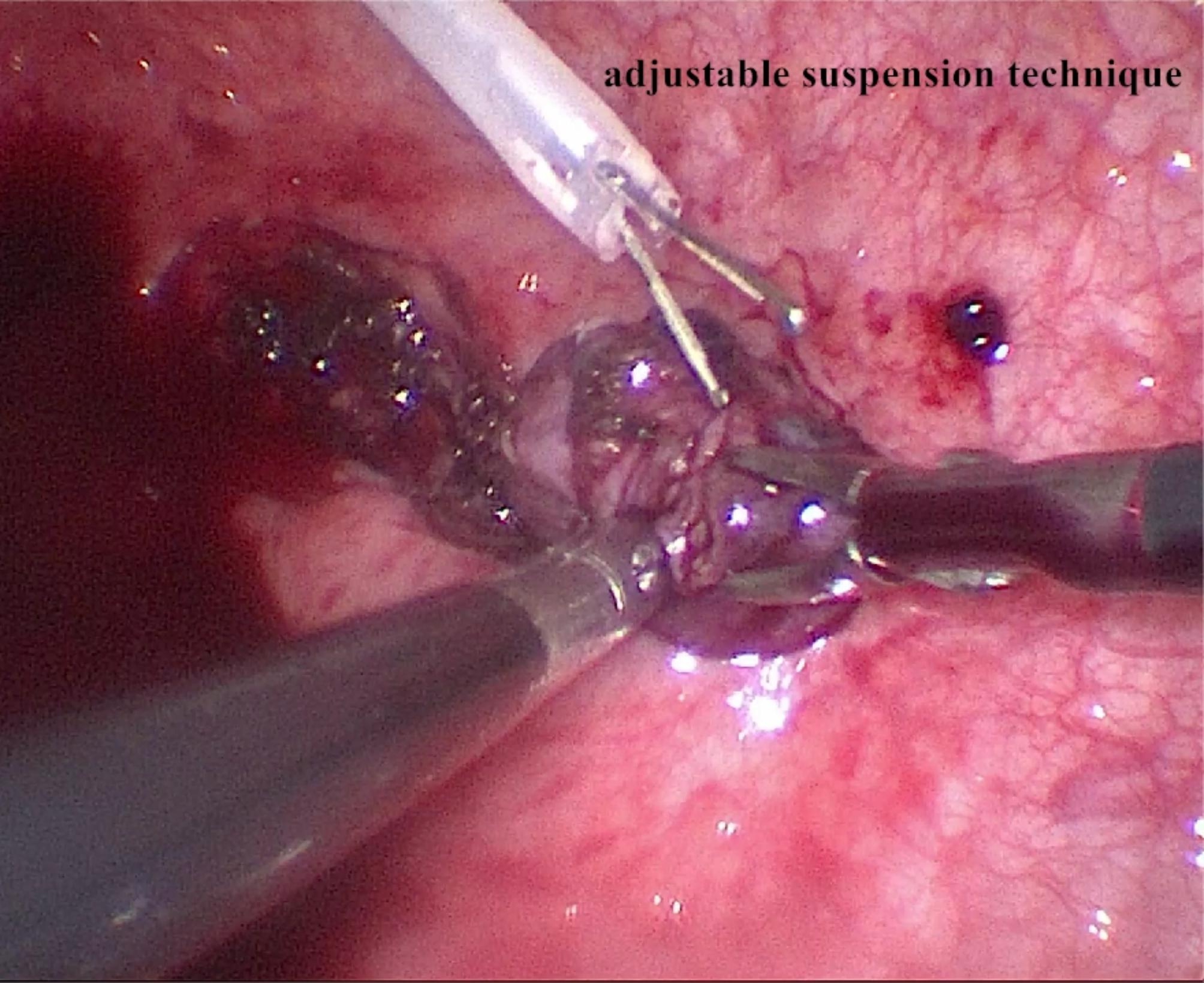
Adjustable suspension technique through urethra assisted pneumovesicoscopic procedures

The pneumoperitoneum pressure was reduced to 12 mmHg. An auxiliary tractor with a diameter of 1.8 mm and a central vent was inserted through the urethra to catch the distal ureter as an “adjustable suspension” (Fig. 2). It can free the operator’s left hand and be beneficial to complete the dissociation of the ureter. Excess urine and fumes can also be eliminated through the central vent. The mucosa was cauterised 0.3 cm around the ureteral orifice with an electric coagulation hook. The muscularis of the bladder wall was carefully detached layer by layer. The objective was to be as close to the ureter wall as possible until the reticular vessels on the ureter surface were found and utilised as a reference plane. The ureter was gradually dissociated into the bladder, and the separating length followed the principle of “5:1”. The ureter with a diameter of more than 2 cm was also trimmed to approximately 0.5 cm. The length was the same as the separating part of the ureter. The ureter was continuously sutured with 5 − 0 absorbable stitches.

The planting distance was measured according to the separating length. Approximately 1.5 cm above the contralateral ureteral orifice was selected as the implantation site. A tunnel was made between the submucosal muscularis with a 3 mm shear. The separating layer should be kept between the mucosa and the muscle layer. For bilateral replantation, a larger diameter ureter that needed to be trimmed was placed upwards. The lower ureter traverses the submucosa of the trigone to the contralateral ureterostoma area. The tunnel should be wide enough to accommodate the ureter and avoid stricture. Sufficient dissociation of the ureter can ensure that the ureter passes through the tunnel without tension and does not form an excessive bending angle. The ureteral opening and bladder mucosa were sutured intermittently with 5 − 0 absorbable string for 4–6 stitches. The ureter has spray urine as evidence of unobstruction. We did not routinely use indwelling double J tubes after surgery.

#### Open cohen surgery

The patient was placed in the supine position. A small transverse arc incision of approximately 5 cm was made in the midline 2 cm above the symphysis pubis. The abdominal subcutaneous tissue and muscle layers were incised to the bladder antetheca. The anterior wall of the bladder was then opened lengthwise. The bladder was filled with gauze to flatten the posterior wall. The ring retractor was used to fully expose the trigone. A stent tube was inserted into the ureter as traction. The methods and principles of ureter and bladder dissociation and reanastomosis were the same as those of pneumovesicoscopic surgery.

### Statistical analysis

SPSS 25.0 software was used for statistical analysis. The quantitative data were expressed as the median [first quartile, third quartile]. The Mann‒Whitney test was used for statistical analysis. The chi-square test or Fisher’s exact test was used to compare the qualitative data between the groups, according to the sample size. A p-value of < 0.05 was defined as statistically significant.

## Result

There were no significant differences in age, sex, weight, degree of preoperative reflux, degree of preoperative hydronephrosis, preoperative glomerular filtration rate, preoperative renal atrophy, preoperative renal scarring, or KIM-1 and MCP-1 expression between the two groups (P > 0.05). (Table 1)

**Table 1:** Comparison of the general data between the two groups

	Pneumovesicoscopic Cohen surgery group	Open Cohen surgery group	P value
Number of patients	31	29	
Number of kidneys	52	49	
Bilateral VUR / unilateral VUR	21/10	20/9	0.919
Age (month)	10 [9, 11]	10 [8.5, 11]	0.706
Males / Female	17/14	17/12	0.768
Weight (kg)	9.5 [8.6, 10.4]	9.8 [8.6, 10.5]	0.636
Grade of VUR			
Grade V	3	5	0.704
Grade IV	23	20	
Grade III	26	24	
Grade II	0	0	
Glomerular filtration rate (%)	50 [43, 59.7]	50 [45, 56.5]	0.634
Renal atrophy	5	3	0.516
Degree of renal scar			
Level 0	45	41	0.856
Level 1	3	2	
Level 2	1	2	
Level 3	3	4	
Anteroposterior diameter of hydronephrosis	0.6 [0.3, 1]	0.5 [0.35, 1.1]	0.835
KIM-1 before operation (ng/mg)	271.2 [248.1, 297.8]	271 [253.2, 299.8]	0.403
MCP-1 before operation (pg/mg)	235.7 [211.0, 256.4]	246.8 [208.4, 284]	0.296

All pneumovesicoscopic Cohen surgeries were successfully completed, and no patients were transferred to open surgery. The amount of bleeding (3 [3, 4] vs. 7 [5, 9.5], p = 0.000), duration of postoperative analgesia (5 [4, 6] vs. 6 [5, 6], p = 0.038), duration of postoperative haematuria (2 [2, 3] vs. 3 [3, 4], p = 0.000), incision size (2 [2] vs. 4 [4, 5], p = 0.001) and length of hospital stay (9 [8, 9] vs. 12 [10, 13], p = 0.000) in the pneumovesicoscopic surgery group were significantly lower than those in the open surgery group. Though the operation time of the pneumovesicoscopic surgery group was significantly longer than that of the open surgery group (unilateral: 123 [113.8, 129.3] vs. 110 [103.5, 119.5], p = 0.022; bilateral: 150 [139, 165] vs. 133 [128, 145], p = 0.001). (Table 2)

**Table 2:** Comparison of clinical perioperational data between the two groups

	Pneumovesicoscopic Cohen surgery group	Open Cohen surgery group	P value
Unilateral duration of surgery (min)	123 [113.8, 129.3]	110 [103.5, 119.5]	0.022
Bilateral duration of surgery (min)	150 [139, 165]	133 [128, 145]	0.001
Amount of bleeding (ml)	3 [3, 4]	7 [5, 9.5]	0.000
Duration of hematuria (d)	5 [4, 6]	6 [5, 6]	0.038
Duration of usage of acesodyne (d)	2 [2, 3]	3 [3, 4]	0.000
Postoperative hospitalization (d)	9 [8, 9]	12 [10, 13]	0.000
Incision length (cm)	2 [2]	4 [4, 5]	0.001

Both groups were followed up 6 months after surgery. At the 6-month follow-up time, there were no significant differences in the degree of hydronephrosis, renal scarring, renal atrophy, glomerular filtration rate, or KIM-1 and MCP-1 expression between the two groups (P > 0.05). There was no recurrence of febrile urinary tract infection or VUR in either group. (Table 3)

**Table 3:** Comparison of postoperative follow-up data between the two groups

	Pneumovesicoscopic Cohen surgery group	Open Cohen surgery group	P value
Anteroposterior diameter of hydronephrosis 6 months after operation	54 [48, 62]	50 [47, 60.5]	0.478
Glomerular filtration rate 6 month after operation	0.5 [0.3, 0.7]	0.5 [0.3, 0.7]	0.329
Renal atrophy 6 months after operation	6	4	0.570
Degree of renal scar 6 months after operation			
Level 0	44	42	0.962
Level 1	2	1	
Level 2	3	3	
Level 3	3	3	
VUR recurrence	0	0	
Febrile urinary tract infection	0	0	
KIM-1 at 6 months after operation(ng/mg)	169 [153.7, 186]	168 [154.2, 176.4]	0.935
MCP-1 at 6 months after operation (pg/mg)	143.1 [122.4, 166.7]	141 [125.8, 178.9]	0.663

## Discussion

Open Cohen surgery has been used in the clinic for many years, and the success rate of surgery is more than 90% 17. However, the issues with open surgery include large trauma, long incision, long postoperative hospital stay, and obvious scars, that cannot be avoided. Since Gill IS et al. 18 and Yeung CK et al. 19 created the innovative pneumovesicoscopic Cohen surgery, other scholars have continuously followed and improved this technique. Kanojia RP et al. 20 combined robotic with pneumaticvesicoscopic technology and applied it to 5 children over 4 years old with a bladder volume of more than 250 ml. The children recovered well after surgery. However, robotics-assisted techniques have limitations, such as limited operating space and high requirements for bladder capacity and the age of children. Trocar diameter is large (8 mm) and is not suitable for infants younger than 1 year old. The equipment is too expensive to be widely promoted in clinical practice.

In the literature, Chang J et al. 21 dissociated the ureter in situ. The bladder muscular layer was directly fixed at the root of the dissociated segment and the distal ureter was allowed to float and form nipples in the bladder. After the removal of the double J tubes, 2 children developed grade I-II reflux. Reflux was relieved spontaneously 3 months later. There were no other complications during the 2-year follow-up period. Benaired AM et al. 22 performed the operation for 60 children with vesicoureteral reflux disease using the pneumaticvesicoscopic Cohen method. There were no postoperative complications. After surgery, they routinely utilized indwelling double J tubes, and postoperative reflux recurred in 3 cases (5%). At present, the technique of pneumovesicoscopic Cohen surgery is relatively mature, but there are few studies on the research and modification of surgical methods specifically for infants. In our centre’s study, we modified the technique of pneumovesicoscopic Cohen surgery and we performed pneumovesicoscopic Cohen surgery with an adjustable suspension technique through the urethra for the treatment of primary vesicoureteral reflux disease in infants. This study summarized our surgical experience. In comparison with the infants who underwent open Cohen surgery, the results showed that pneumovesicoscopic Cohen surgery with an adjustable suspension technique through the urethra was safe and effective and had the advantages of less trauma, quick recovery, and aesthetic appearance.

The application of pneumovesicoscopic Cohen in infant patients also has its disadvantages. For example, the operating space in infants is a much smaller area than that of children over 1 year of age. The surgeon needs to perform the operation nearly vertically. The chopstick effect is much more obvious. Bilateral replantation is more difficult and requires a significant learning curve. The experience of pneumovesicoscopic Cohen surgery with an adjustable suspension technique through the urethra is summarized as follows: (1) Trocar placement: We initially had high intra-bladder pneumoperitoneum pressure, and due to the thinner and softer muscle layer of the bladder in infants, the failure rate of trocar insertion is relatively high. We reserve a suspension suture to fit the bladder to the abdominal wall so that the risk of puncture failure is greatly reduced. The puncture site is usually located in the anterior wall of the bladder. A higher puncture position may cause a false path and gas leakage into the abdominal cavity. The knot must be tightened after successful puncture. (2) Transurethra tractor-assisted technique (adjustable suspension technology): A very fine auxiliary gripper with a central orifice is introduced through the urethra into the bladder. This procedure may eliminate excess electrocoagulation smoke, urine, and blood without additional trauma and maintain clear vision during surgery. At the same time, it can be used as traction equipment for ureter dissociation in patients with megaureters, obvious edema, and fibrosis in the inner segment of the bladder wall after repeated infection. The suspension position can be adjusted by swinging the grasping pliers by the assistant, which can lead to better surgical exposure and a prosperous dissociating procedure. It also eliminates the need to insert a stent tube into the ureter for lifting and shortens the operating time.

Because the bladder space of younger age and weight patients was very limited, the operation was extremely difficult, the surgical instruments interfered with each other significantly, and the distance of ureter implantation cannot be guaranteed. These difficulties may lead to high postoperative complications and surgical risks. In our centre, for children of younger age and weight (age < 6 months or weight < 7 kg), we did not perform pneumovesicoscopic Cohen surgery, and all children underwent open Cohen surgery.

It is important to also discuss the study’s limitations. First, this study is a retrospective analysis with a small number of samples. Second, this study is a single-centre study, and additional studies are needed to confirm the effectiveness and complication rate of this modified surgical method. Third, the follow-up time of this study is not quite long enough. A longer follow-up is needed to evaluate long-term renal scar formation and renal function influence after pneumovesicoscopic surgery.

## Conclusion

Pneumovesicoscopic Cohen surgery with an adjustable suspension technique through the urethra for the treatment of primary vesicoureteral reflux disease in infants was safe and effective. Its advantages included less trauma, quick recovery and good cosmetic effects. The use of an adjustable suspension technique through the urethra can reduce the difficulty of minimally invasive surgery, which can be better applied to infants.

## Data Availability

The datasets of the current study are available from the corresponding author upon. reasonable request.

## Abbreviations

VUR: primary vesicoureteral reflux
